# Molecular Characterisation of Soybean Osmotins and Their Involvement in Drought Stress Response

**DOI:** 10.3389/fgene.2021.632685

**Published:** 2021-06-25

**Authors:** Giulia Ramos Faillace, Paula Bacaicoa Caruso, Luis Fernando Saraiva Macedo Timmers, Débora Favero, Frank Lino Guzman, Ciliana Rechenmacher, Luisa Abruzzi de Oliveira-Busatto, Osmar Norberto de Souza, Christian Bredemeier, Maria Helena Bodanese-Zanettini

**Affiliations:** ^1^Programa de Pós-Graduação em Genética e Biologia Molecular and Instituto Nacional de Ciência e Tecnologia: Biotec Seca-Pragas, Departamento de Genética, Instituto de Biociências, Universidade Federal do Rio Grande do Sul (UFRGS), Porto Alegre, Brazil; ^2^Laboratório de Bioinformática, Modelagem e Simulação de Biossistemas (LABIO), Pontifícia Universidade Católica do Rio Grande do Sul (PUCRS), Porto Alegre, Brazil; ^3^Programa de Pós-Graduação em Biologia Celular e Molecular, Pontifícia Universidade Católica do Rio Grande do Sul (PUCRS), Porto Alegre, Brazil; ^4^Programa de Pós-Graduação em Fitotecnia, Departamento de Plantas de Lavoura, Faculdade de Agronomia, Universidade Federal do Rio Grande do Sul (UFRGS), Porto Alegre, Brazil; ^5^Programa de Pós-Graduação em Biologia Celular e Molecular, Centro de Biotecnologia (CBiot), Universidade Federal do Rio Grande do Sul (UFRGS), Porto Alegre, Brazil

**Keywords:** phylogenetic analysis, gene structure, chromosomal localisation, protein structure, gene expression, response to dehydration

## Abstract

Osmotins are multifunctional proteins belonging to the thaumatin-like family related to plant stress responses. To better understand the functions of soybean osmotins in drought stress response, the current study presents the characterisation of four previously described proteins and a novel putative soybean osmotin (GmOLPa-like). Gene and protein structure as well as gene expression analyses were conducted on different tissues and developmental stages of two soybean cultivars with varying dehydration sensitivities (BR16 and EMB48 are highly and slightly sensitive, respectively). The analysed osmotin sequences share the conserved amino acid signature and 3D structure of the thaumatin-like family. Some differences were observed in the conserved regions of protein sequences and in the electrostatic surface potential. P21-like present the most similar electrostatic potential to osmotins previously characterised as promoters of drought tolerance in *Nicotiana tabacum* and *Solanum nigrum*. Gene expression analysis indicated that soybean osmotins were differentially expressed in different organs (leaves and roots), developmental stages (R1 and V3), and cultivars in response to dehydration. In addition, under dehydration conditions, the highest level of gene expression was detected for GmOLPa-like and P21-like osmotins in the leaves and roots, respectively, of the less drought sensitive cultivar. Altogether, the results suggest an involvement of these genes in drought stress tolerance.

## Introduction

Due to plants being sessile organisms, they are often exposed to various abiotic stresses such as drought, cold, and soil salinity. These environmental stresses result in osmotic changes that can lead to the disruption of normal cellular activities, thus affecting plant growth and development. In crop plants, these effects could also hamper productivity (Husaini and Rafiqi, 2012). To avoid the consequences of multiple stresses, plants have evolved highly complex and sophisticated defence mechanisms such as the production of various defence-related proteins known as pathogenesis-related (PR) proteins. PRs are classified into 17 families (PR1 to PR17) based on their amino acid composition, structure, and biochemical function (Misra et al., 2016).

Osmotins are stress responsive multifunctional proteins belonging to the Pathogenesis-related proteins family 5 (PR5) (also known as the thaumatin-like family) due to their high sequence similarity to thaumatin, a sweet-tasting protein from the West African shrub *Thaumatococcus danielli* (Abdin et al., 2011). Osmotins were first isolated and characterised in tobacco cells adapted to a low osmotic potential, but also have been induced in several plant species - including *Glycine max* (soybean) - in response to various abiotic and biotic stresses (Parkhi et al., 2009; Weber et al., 2014). In view of their involvement in stress responses, many studies have aimed to overexpress osmotin in agronomically important crops (Barthakur et al., 2001; Parkhi et al., 2009; Goel et al., 2010; Annon et al., 2014; Weber et al., 2014). Transgenic plants overexpressing osmotin-like proteins have been shown to confer tolerance to salt, drought, freezing, as well as fungal and bacterial infection (Kumar S. A. et al., 2016).

As a primary contributor to global food production, soybean is one of the most important commodities. Current and predicted climate change, which suggests increased frequency, duration, and severity of drought periods, or intense heat, represent a serious challenge for agricultural production in Brazil and worldwide (Cunha et al., 2014; Chen et al., 2016). The evaluation of the relative effect of climate and agricultural technology on soybean productivity in Brazil indicated that some regions can be more heavily affected by climate change. Although the environmental suitability of some areas would increase, an overall decrease in environmental suitability was observed, indicating that soybean cultivation in Brazil could be highly threatened in the future (Caetano et al., 2018).

Drought is one of the most relevant environmental factors that dramatically limits soybean grain yield. Over the years, the development of drought-tolerant cultivars has served as a solution to yield losses (Shin et al., 2015). Moreover, a promising strategy to develop tolerance against abiotic stress is based on the overexpression of PR proteins - such as osmotins - in transgenic plants (Ahmed et al., 2013). Notably, Weber et al. (2014) demonstrated that the expression of *Solanum nigrum* osmotin in soybeans improved the physiological responses and yield components of transgenic plants subjected to water deficit. However, the molecular basis of osmotin action in response to drought remains unclear. In this sense, any attempt to improve stress tolerance first requires an improved understanding of the underlying physiological, biochemical, and molecular events (Abdin et al., 2011).

To date, soybean presents four identified osmotins: soybean leaf polypeptide (P21), *Glycine max* osmotin-like protein, acidic isoform (GmOLPa), *Glycine max* osmotin-like protein, basic isoform (GmOLPb), and P21-like protein from *Glycine max* (L.) Merr. cultivar Enrei (P21e). P21 protein was the first osmotin identified in soybean (*Glycine max* var Williams 82), and has been purified from mature leaves without any stress treatments, although authors suggest that the protein could be accumulated as a response to infection and stress (Graham et al., 1992). GmOLPa, GmOLPb, and P21e isoforms were further characterised as being involved in high-salt stress and hormonal responses in *Glycine max* (L.) Merr. cultivar Enrei from Northern blot analysis (Onishi et al., 2006; Tachi et al., 2009). Despite their high sequence similarities, some differences in three-dimensional (3D) structure, electrostatic potential, subcellular location, and gene expression suggested that each soybean osmotin could play a distinctive role in defence against high salt stress (Tachi et al., 2009). Although three soybean osmotins have been suggested as being related to salinity response, the roles of soybean osmotins in drought stress and tolerance remain unclear.

Aiming to contribute to the understanding of soybean osmotins role in drought stress response, in the present study several analyses were performed. The analyses included the protein sequence, subcellular location, individual 3D protein structure, electrostatic potential, gene structure, chromosomal position, gene duplication, putative cis-elements, and gene expression pattern in different tissues and developmental stages of two soybean cultivars with different sensitivities to dehydration. Due to its sensibility the RT-qPCR tool was chosen for the osmotin encoding-genes expression analysis.

## Materials and Methods

### Data Mining and Phylogenetic Analyses

Data mining was performed to identify osmotins that have been expressed in transgenic plants and have demonstrated a relationship to drought tolerance. Previously identified osmotins from *Nicotiana tabacum* (X61679) (Das et al., 2011) and *Solanum nigrum* (AF450276) (Weber et al., 2014) were used as queries for a blastp search against the *Glycine max* full genome available at Phytozome v.012. Two other previously characterised *S. nigrum* osmotins (AF473702 and KC292261) were also included in the analysis. All soybean sequences retrieved from blastp share the thaumatin domain, thus suggesting that they belong to the thaumatin-like family. Protein isoforms were excluded to refine the analysis. The full-length coding sequences (cds) were translated into amino acid sequences and aligned using the Muscle algorithm from MEGA7 (Molecular Evolutionary Genetics Analysis) software (Kumar S. et al., 2016). The thaumatin domain sequences of *N. tabacum* and *S. nigrum* were identified by the Simple Modular Architecture Research Tool (SMART) (Letunic and Bork, 2017) and used as a reference to determine the thaumatin domain region of the other aligned sequences. The thaumatin domain sequence was then used for phylogenetic analysis. The sequences were manually edited, and the Prottest 3.4 program (Abascal et al., 2005) was used to identify the optimal protein evolution model for Bayesian analysis. The phylogenetic analysis was reconstructed using the Bayesian method in the Beast 1.8 package (Drummond et al., 2012). WAG+G was the best model for protein sequences dataset according Prottest (Abascal et al., 2005). The Birth-Death process was selected as a tree prior to Bayesian analysis, and 50,000,000 generations were performed with the Markov Chain Monte Carlo (MCMC) algorithms. Tracer 1.6 (Rambaut et al., 2014) was used to verify the effectivity of obtained data by the convergence of Markov chains and adequate effective sample sizes (>200) after the first 10% of generations had been deleted as burn-in. The TreeAnnotator (Beast 1.8 package) was used to access the maximum clade credibility of the consensus tree. The tree was visualised and edited using FigTree v1.4.3^1^. Statistical support for the clades was determined by accessing the Bayesian posterior probability. Further editions were performed by visual analysis of sequence organisation in the reconstructed tree.

### Gene and Protein Structure Analysis

Osmotin sequences were analysed for gene and protein structures as well as signal peptide information. Intron/exon structures and organisation were determined from the Gene Structure Display Server (GSDS) program, developed by the Centre of Bioinformatics (CBI), Peking University (Hu et al., 2015). The protein structures, signal peptides, and subcellular locations were predicted using SMART (Letunic and Bork, 2017), SignalP 4.1 Server^2^, and TargetP 1.1 Server^3^, respectively. To verify the conserved residues, protein sequences were aligned using MEGA 7 software, and the alignments were further visually inspected using GeneDoc (Nicholas et al., 1997). The conserved residues were identified according to Petre et al. (2011) and Ahmed et al. (2013).

### Chromosomal Localisation and Duplication Pattern

The chromosomal localisation and gene duplication patterns were determined for soybean osmotin sequences using the Genome Duplication Database (PGDD), considering a 100kb syntenic region (Lee et al., 2013) and MCScanX software (Wang et al., 2012).

### Bioinformatic Sequences Analysis and Comparative Modelling Protocol

The SignalP 4.1 sequence analysis (Petersen et al., 2011) revealed that the first 20 amino acids were identified as a signal peptide. Hence, these residues were excluded from the molecular modelling procedure. We used the comparative modelling approach, and implemented in the MODELLER 9v19 program (Sali and Blundell, 1993) to construct 3D models of osmotins Gma_OLPb, Gma_P21like, Gma_P21, Gma_GmOLPa-like, and Gma_OLPa based on the 3D structure of grape thaumatin-like protein (PDB ID 4L5H) (Marangon et al., 2014). For Sni_SnOLP, Sni_SindOLP and Sni_Jami models were based on the 3D structure of NP24-I from *Solanum lycopersicum* (PDB ID: 2I0W) (Ghosh and Chakrabarti, 2008).

The protocol used to perform molecular modelling experiments was: generation of 10 models, from which one model for each osmotin sequence was selected. All models were submitted to the DOPE energy scoring function (Shen and Sali, 2006) implemented in MODELLER 9v19 to select the best structures. The MOLPROBITY webserver (Chen et al., 2010) and PROCHECK (Laskowski et al., 1993) were used to verify and validate the stereochemical quality of the models. Electrostatic surface potential were calculated using the program APBS and displayed with the PyMOL program (The PyMOL Molecular Graphics System, Version 1.5.0.4 Schrödinger, LLC). All images were generated using the PyMOL program. Multiple sequence alignment comparisons were performed using ClustalW (Combet et al., 2000) using the Blosum matrix for amino acid substitutions and the default parameters to infer possible structural similarities.

### Gene Expression Data Mining

In order to gain insights regarding gene expression, soybean osmotin RNA-seq expression data were searched in the Soybean eFP Browser^4^ (Libault et al., 2010), RNA-Seq Atlas of *Glycine max* (Severin et al., 2010), and data from drought stress experiments (Le et al., 2012; Belamkar et al., 2014; Shin et al., 2015; Chen et al., 2016).

### Soybean Dehydration Assay for Gene Expression Analysis

To improve the investigation on soybean osmotin expression in response to drought stress, two experiments were performed using the BR16 and EMBRAPA48 (EMB48) soybean cultivars, which are highly and slightly sensitive to dehydration stress (Oya et al., 2004), respectively.

The first experiment was performed under greenhouse conditions at an air temperature of 28 ± 5°C using natural illumination. Plants were grown in 5L-plastic pots filled with a substrate/soil mixture. Eight seeds were sown per pot, and four plants remained after thinning following plant emergence. Prior to sowing, seeds were inoculated with two *Bradyrhizobium* strains (*B. elkanii* and *B. japonicum*). The inoculation reflects the way in which soybean is usually produced in the field. The biological N2 fixation (BNF) is one of the processes of the soybean plant most sensitive to water deficiency. Although BNF may be inhibited by water deficit, there is genetic variability in this response, which may be responsible for the differential tolerance to water stress between legumes species and between soybean genotypes (Sall and Sinclair, 1991; Serraj et al., 1999; Daryanto et al., 2015; Cerezini et al., 2019). Once a week, a complete Hoagland nutrient solution (nitrogen-free) was applied to each pot. Treatments consisted of two water regimes (control/irrigated and drought stress/non-irrigated), and were imposed for 38 days after sowing, when plants were at R1 growth stage (onset of flowering, beginning flowering - plants have at least one flower on any node) (Fehr and Caviness, 1977). From plant emergence until the R1 growth stage, pots were weighed daily and irrigated with water (if necessary) to maintain soil moisture at approximately 90% of field capacity. The experiment was performed using a randomised block design with four biological replicates. Each pot was considered an experimental unit. In order to characterise drought stress and physiological responses, relative leaf water content (RWC), minimum leaf water potential, leaf temperature, and the quantum yield of photosystem PSII were determined at 8 (moderate stress) and 10 (severe stress) days after watering suspension. The uppermost fully expanded leaves were used for data collection. Leaves from both cultivars with four biological replicates were also collected and frozen in liquid nitrogen for gene expression analysis. Samples were stored at −80°C until the analyses were performed.

In the second experiment, plants from the two cultivars (BR16 and EMB48) were grown in pots containing vermiculite supplemented with half strength MS medium (Murashige and Skoog Basal Salt Mixture) once per week. The pots were maintained under growth chamber conditions (26 ± 1°C with a 16/8 h light/dark cycle at a light intensity of 22.5 μE m^−2^ *s*^−1^) and irrigated once per day. At the V3 stage (third trifoliolate - three sets of unfolded trifoliolate leaves), plants were removed from pots, roots were washed, and entire plants were exposed to air. Leaves and roots were collected from both cultivars with five biological replicates at three time points (0, 6, and 12 hours). Samples were frozen in liquid nitrogen and stored at −80°C.

### Physiological Analysis

Relative leaf water content (RWC) estimates the current water content of sampled leaf tissue relative to the maximum water content it can hold at full turgidity, which was determined by the following relationship: RWC(%) = [(fresh weight – dry weight)/(turgid weight – dry weight)]^∗^100. Firstly, the fresh weight of two leaves per plant and per treatment was determined. Thereafter, leaves were left floating in distilled water in Petri dishes for 24 h, and the turgid weight was then recorded. Finally, leaves were dried in an oven at 65°C for 48 h, and their dry weight was measured (Salvador et al., 2012). Minimum leaf water potential was measured at 1:00pm using a Scholander-type pressure chamber (Model 3000, Soil Moisture Co., EUA) (Scholander et al., 1965; Boyer, 1967).

Leaf temperature was measured remotely by an infrared thermometer (Incoterm Co., São Paulo, Brazil) with temperature range of −10 to 60°C, emissivity of 0.98 W m^–2^, and a field of view of 2.80°. Readings were performed at the same time (11:00am) and at same distance (15 cm) between the leaf and thermometer on the adaxial surface of the uppermost fully expanded leaf in four plants per replicate. At this distance, the measured area on leaf surface is approximately 9 mm^2^. During measurements, air temperature was recorded, and the difference between both variables (ΔT) was calculated by the following relationship: ΔT_*leaf* – air_.

The quantum yield of photosystem II was determined under natural light conditions with a portable pulse modulation fluorometer (Model OS1-FL, Opti-Sciences, Hudson, NY, United States). Measurements were performed at 11:00am on the adaxial surface of the uppermost fully expanded leaf in four leaves per replicate.

### Gene Expression Analysis

Total RNA was extracted with a Trizol reagent (ThermoScientific) and treated with DNase I (ThermoScientific) according to the manufacturer’s instructions. First-strand cDNAs were obtained using 1 μg of DNA-free RNA, M-MLV Reverse Transcriptase System^TM^ (ThermoScientific) and oligo(dT) primers. To evaluate relative gene expression, the first-strand cDNA reaction product was diluted at 1:100.

Gene expression was analysed using real-time quantitative polymerase chain reaction (RT-qPCR). Primers were designed using Primer3 software^5^ (Koressaar and Remm, 2007; Untergasser et al., 2012) according to the gene sequences (Supplementary Table 1). The reactions were performed in a 25 μL volume containing 10 μM of each primer, 12.5 μL of diluted cDNA sample (1:100), 1 × PCR buffer, 50 mM MgCl_2_, 10 mM of each dNTP, 2.5 μL SYBR-Green solution (1:100,000, Molecular Probes Inc., Eugene, OR, United States), and 0.06 U Platinum Taq DNA Polymerase (ThermoScientific). The RT-qPCR was performed using a StepOne Applied Real-Time Cycler in a 96-well plate. Cycling conditions were implemented as follows: 5 min at 94°C for an initial denaturation, 40 cycles of a 10 s denaturation step at 94°C, a 15 s annealing step at 60°C, and a 15 s extension step at 72°C ending with 2 min at 72°C for a final extension. Melting curve analysis was performed at the end of the PCR run over a range of 55–99°C, increasing the temperature stepwise by 0.1°C/s. Technical triplicate reactions were performed for each sample. The CYP2 and ELF1A genes were used as references for expression normalisation. Relative expression fold changes were determined using the 2^–ΔΔ*Ct*^ method described by Livak and Schmittgen (2001).

### Statistical Analysis

Physiological data from the two soybean cultivars, which were submitted or not to drought stress at the R1 reproductive stage, were subjected to analysis of variance (ANOVA). When the *F*-test was significant (*p* < 0.05), a comparison of means was performed by Duncan’s test (*p* = 0.05) using the software SPSS Statistics version 17.0. Gene expression data were subjected to a log_2_ transformation prior to analysis in order to make data distribution more symmetrical (less skewed), since log-transformed data have less extreme values compared to untransformed data (Willems et al., 2008; Zwiener et al., 2014).

### *In silico* Promoter Analysis

The putative promoter region - located 2,000 base pairs (bp) upstream of the transcription start site (TSS) of each soybean osmotin gene - was used to search for putative *cis*-elements. The analysis was performed using the Plant Pan Database^6^ (Chang et al., 2008). In addition, the transcript factors (TFs) identified by Plant Pan Database were searched in the RNA-seq data available from the literature on soybean submitted to drought stress. Only upregulated TFs genes were included.

## Results

### Data Mining and Phylogenetic Analysis

Transgenic plants of different species expressing *N. tabacum* and *S. nigrum* osmotin genes have been shown to exhibit enhanced drought stress tolerance (Table 1). Two previously characterised osmotins (Nta_X61679_OSM and Sni_AF450276_SnOLP) were used as query sequences for blastp in soybean genome. A total of 61 soybean sequences were retrieved. The four *N. tabacum* and *S. nigrum* osmotin sequences (Nta_X61679_OSM, Sni_AF450276_SnOLP, Sni_AF473702_Jami, and Sni_KC292261_SindOLP) (Table 1) were included in further analyses. Following protein alignment, six sequences showed missing data, and therefore were excluded.

**TABLE 1:** Transgenic plants overexpressing osmotins that confer drought tolerance.

Gene name	ID	Donor	Transgenic plant	References
Osmotin	-	*Nicotiana tabacum*	*Nicotiana tabacum*	Barthakur et al. (2001)
	-		*Gossypium hirsutum L.*	Parkhi et al. (2009)
	-		*Solanum lycopersicum*	Goel et al. (2010)
	X61679		*Morus indica*	Das et al. (2011)
	-		*Daucus carota L.*	Annon et al. (2014)
	M29279		*Camellia sinensis L.*	Bhattacharya et al. (2014)
	-		*Olea europaea L.*	Silvestri et al. (2017)
*SnOLP*	AF450276	*Solanum nigrum*	*Glycine max*	Weber et al. (2014)
*OLP*	AF473702		*Solanum lycopersicum*	Kumar S. A. et al. (2016)
*SindOLP*	KC292261		*Sesamum indicum*	Chowdhury et al. (2017)

*”-” indicates that the sequence ID was not available in the manuscript.*

**FIGURE 1:**
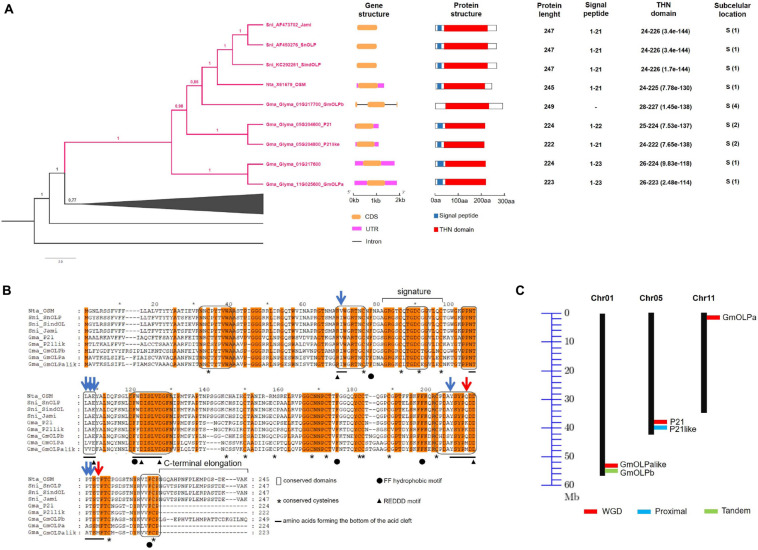
Osmotin *in silico* analysis. **(A)** Detailed representation of the phylogenetic relationship among the thaumatin domain within the osmotin monophyletic clade with corresponding gene and protein structure information. According to the phylogenetic results the Gma_Glyma_01G217600 protein was named GmOLPa-like. Subcellular locations are numbered according to the TargetP server from one to five, where one indicates the strongest prediction. S, secretory pathway. **(B)** Alignment of osmotin protein sequences. Conserved residues and protein secondary structure are indicated according to Petre et al. (2011). *Orange* letter background corresponds to conserved amino acids (aa) among the nine analysed sequences. *Blue* and *red* arrows indicate aa substitutions with similar and different properties, respectively. **(C)** Chromosomal location and duplication of soybean osmotins. *Black* vertical lines represent the chromosomes with their numbers at the top. WGD, whole-genome duplication.

A phylogenetic tree reconstructed with the remaining 59 thaumatin-like sequences allowed the identification of an osmotin monophyletic clade including the eight previously identified osmotins from *N. tabacum*, *S. nigrum*, *G. max* (Nta_OSM, Sni_SnOLP, Sni_SindOLP, Sni_Jami, Gma_01G217700_GmOLPb, Gma_05G204600_P21, Gma_05G204800_P21-like, and Gma_11G025600_GmOLPa), and a fifth soybean osmotin (Gma_Glyma_01G217600) that has yet to be characterised (Supplementary Figure 1). Gma_Glyma_01G217600 was identified as a homologous sequence of GmOLPa and named GmOLPa-like (Figure 1A). A subclade for the Solanaceae sequences (*N. tabacum* and *S. nigrum*) was formed inside the osmotin group. Moreover, a common ancestor was shared by the Solanaceae subclade and the GmOLPb sequence. Furthermore, the homologous sequences P21 and P21-like share a common ancestor with GmOLPb and the Solanaceae subclade, indicating more similarities among these sequences than GmOLPa and GmOLPa-like (Figure 1A).

### Gene and Protein Structure

In order to identify similarities between the osmotins, gene and protein structures were analysed for exon-intron organisation pattern, protein length, domain, signal peptide, and conserved residues.

Gene and protein structures were very similar among osmotins, which generally have no introns along the gene sequence and present a signal peptide followed by the thaumatin domain (Figure 1A). GmOLPb was the unique exception, presenting two introns in its sequence and no signal peptide. Osmotin protein length varied from 222 (P21like) to 249 (GmOLPb) amino acids (aa). All osmotin proteins presented a thaumatin domain, and are predicted to be targeted to the secretory pathway, although GmOLPb had the second lowest classification (4 of a range 1-5). The three *S. nigrum* osmotins presented the same gene and protein structures, THN domain, and signal peptide length (Figure 1A).

In general, the conserved amino acids described for the thaumatin domain – 16 cysteine residues, REDDD, and FF hydrophobic motifs – were identified in the osmotin sequences in similar positions to those described in previous publications (Petre et al., 2011; Ahmed et al., 2013), with the exception of Nta_OSM – which lost a cysteine residue – and for GmOLPa-like, which presents the aspartate^109^ residue (D) instead of the glutamate^109^ (E) in the REDDD motif (Figure 1B). Despite this change, the two amino acids (D and E) have the same characteristics (polar, hydrophilic, and negatively charged), implying a substitution without different properties. Other amino acid substitutions were observed in the acid cleft position, some of which between amino acids with similar properties and others with different properties. For example, the substitution of methionine^210^ (M) in GmOLPa and GmOLPa-like by lysine^210^ (K) in P21 and P21-like, and the substitution of glutamine^210^ (Q) in Nta_OSM, Sni_SnOLP, Sni_SindOLP, Sni_Jami, and GmOLPb. Methionine residue is hydrophobic and neutral, while lysine is hydrophilic, polar, and negatively charged, and glutamine is hydrophilic, polar, and neutral. Other substitutions between amino acids with different properties were observed in the methionine^216^ (M) of GmOLPa-like and GmOLPa sequences. This residue was replaced by a threonine^216^ (T) in the other osmotins, which is a polar, hydrophilic, and neutral amino acid (Figure 1B).

### Chromosomal Localisation and Duplication Pattern

**FIGURE 2:**
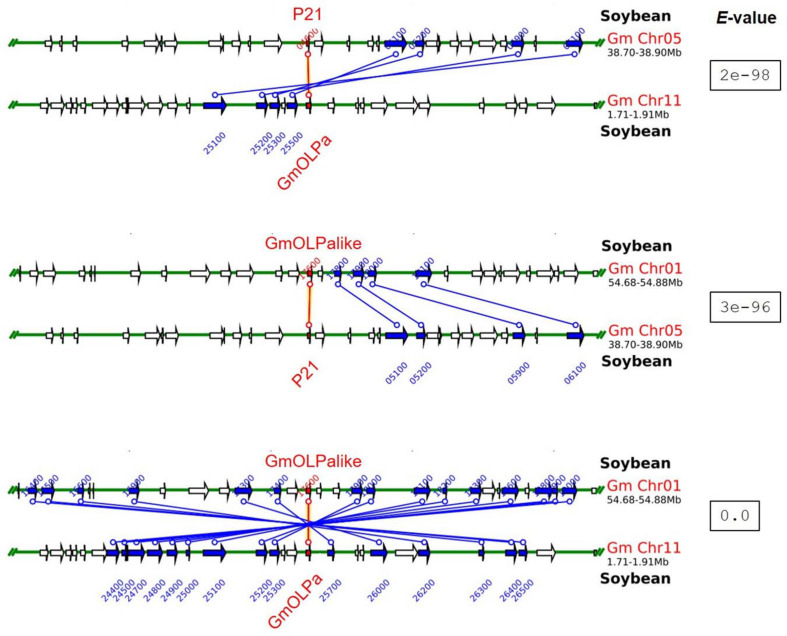
Syntenic regions between paralogous soybean osmotin genes. *Green* horizontal lines represent the chromosomes. *Blue* and *red* vertical lines and arrows represent the duplicated paralogous genes. The *red* lines represent the sequence used as the search query (indicated at the top of each syntenic region). The *white* arrow indicates no duplicated genes. The *e*-value for each syntenic region is shown.

The chromosomal localisation of soybean osmotin-encoding genes and the putative mechanism of their duplication are illustrated in Figure 1C. The soybean osmotin-encoding genes are distributed on chromosomes 1, 5, and 11. On chromosome 1, two osmotin genes are identified (GmOLPa-like and GmOLPb), which are classified as WGD (whole genome duplication) and tandem duplication. P21 and P21-like, WGD and proximally duplicated, respectively, are localised on chromosome 5. In addition, the osmotin gene GmOLPa was identified on chromosome 11 and classified as WGD. All soybean osmotin genes were localised at the end of chromosomes (Figure 1C). A synteny analysis confirmed the WGD classification of the paralogous soybean osmotin genes GmOLPa-like, P21, and GmOLPa (Figure 2).

### Protein Sequence Analysis and Comparative Modelling

Although significant differences have not been observed in amino acid content, sequence analysis of osmotins revealed differences in net charge. The net charges of *S. nigrum* (Sni_SnOLP, Sni_SindOLP, and Sni_Jami) and *N. tabacum* (Nta_OSM) proteins ranged from 0 to +5, whereas those of *G. max* (GmOLPa, Gma_P21like, Gma_P21, Gma_GmOLPa-like, and Gma_OLPb) varied from −5 to +4 (Table 2). Notably, only the Gma_P21-like osmotin has a positive net charge (+4), which is similar to the cationic osmotins of *S. nigrum* and *N. tabacum*.

**TABLE 2:** Glycine, proline, and threonine amino acids content in *Nicotiana tabacum*, *Solanum nigrum*, and *Glycine max* osmotin sequences.

	Glycine	Proline	Threonine	Net charge
Nta_OSM	11.4 %	8.2 %	9.4 %	5
Sni_SnOLP	10.5 %	8.5 %	10.1 %	0
Sni_SindOLP	10.9 %	8.5 %	9.7 %	2
Sni_SnJami	10.5 %	8.5 %	9.7 %	0
Gma_GmOLPb	12.0 %	7.6 %	7.2 %	−1
Gma_P21like	10.8%	6.3%	9.5%	4
Gma_GmP21	12.1 %	6.2 %	8.5 %	−1
Gma_GmOLPa-like	9%	5.8%	10.3%	−4
Gma_GmOLPa	9.8 %	5.8 %	9.4 %	−5

**FIGURE 3:**
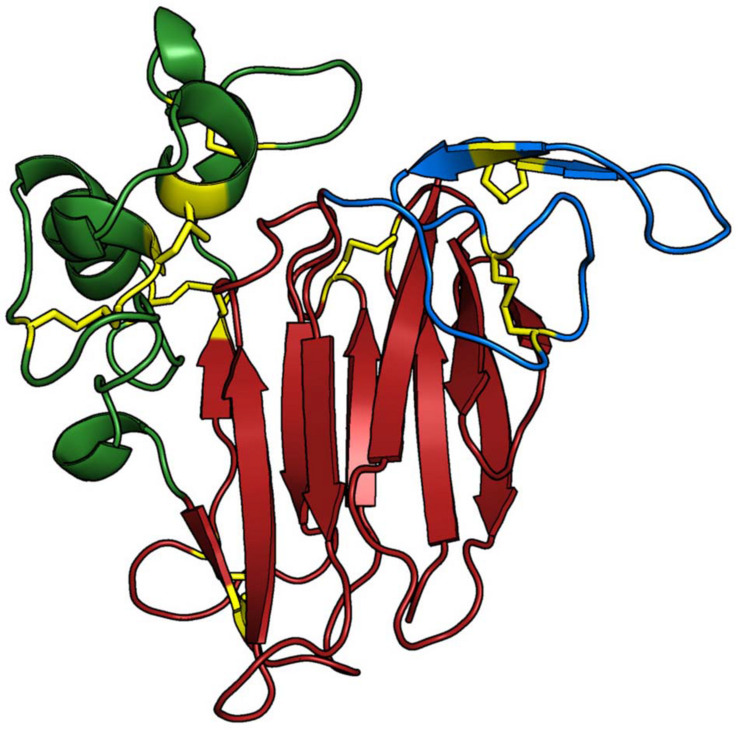
Three-dimensional structure of Gma_P21. The protein structure is coloured based on its domains: (i) domain I (*red*), consisting of a 11 stranded β-sheet organised as a β-barrel that forms the protein core; (ii) domain II (*green*), consisting of an α-helix and a set of disulphide-rich loops; and (iii) domain III (*blue*), presenting a β-hairpin and a coil region, which are both maintained by one disulphide bond each. The main chain of the Osmotin structure is represented as cartoon and the side-chains of the residues involved in disulfide bonds are presented as yellow sticks. The Sni_SnOLP, Sni_SindOLP, Sni_Jami, Gma_OLPb, Gma_P21like, Gma_P21, Gma_OLPc, and Gma_OLPb structures share the same topology. The image was generated with PyMOL Molecular Graphics System version 1.5.0.4 (Schrödinger, LLC).

In order to characterise differences among the osmotins of *N. tabacum*, *S. nigrum*, and *G. max*, three-dimensional (3D) structures for all proteins were built. The analysis revealed a conserved overall architecture of the osmotin proteins Nta_OSM, Sni_SnOLP, Sni_SindOLP, Sni_Jami, Gma_OLPa, Gma_GmOLPa-like, Gma_P21, Gma_P21-like, and Gma_OLPb, which is also preserved among the thaumatin protein family (Anzlovar and Dermastia, 2003). The modelled structures comprise three domains: (i) domain I, containing 11 stranded β-sheets organised as a β-barrel, forming the protein core; (ii) domain II, containing an α-helix and a set of disulphide rich-loops; and (iii) domain III, containing a β-hairpin and a coil motif, both maintained by a disulphide bond (Figure 3).

**FIGURE 4:**
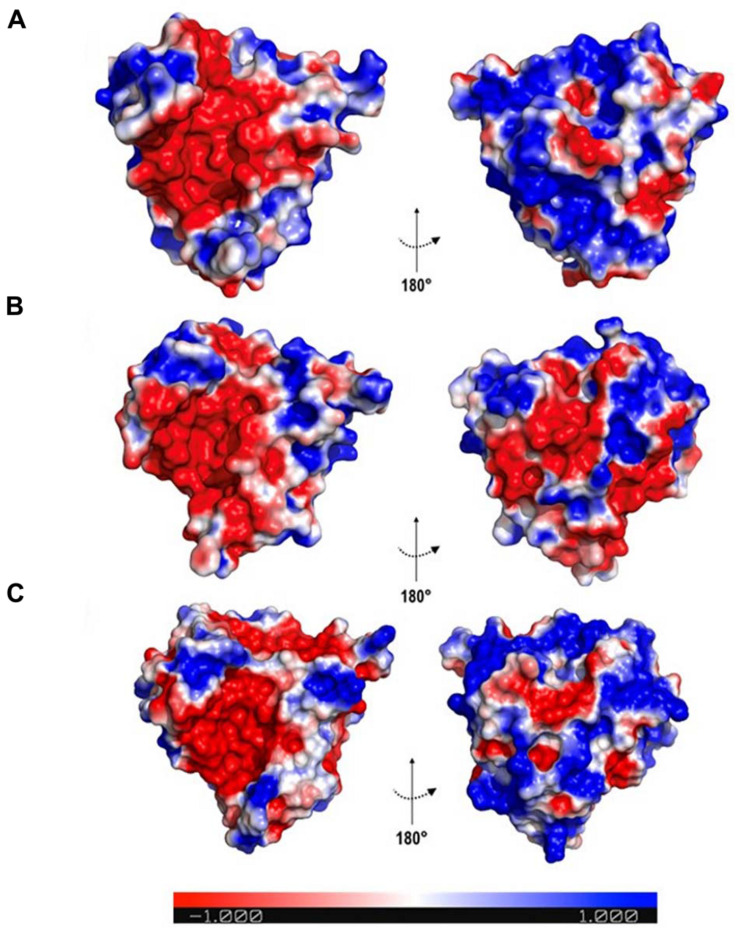
Electrostatic surface potential of osmotin proteins. The colours indicate charges in the electrostatic surface potential; where *red, blue*, and *white* represent negative, positive, and neutral regions, respectively. **(A)** Sni_SnOLP image representing the electrostatic surface potential of *Solanum nigrum* and *Nicotiana tabacum* osmotins. **(B)** Gma_OLPb image representing the electrostatic surface potential of *Glycine max* osmotins, except Gm_P21-like. **(C)** Gma_P21-like. Images generated with PyMOL Molecular Graphics System version 1.5.0.4 (Schrödinger, LLC).

Since the fold is conserved, the distribution of charges in the protein surface was evaluated based on the electrostatic potential surface, which aimed to identify different patterns among proteins. It was observed that all osmotins displayed a negatively charged cavity (Figure 4). However, the *N. tabacum* and *S. nigrum* proteins presented a predominantly positively charged posterior region, whereas for *G. max* that region is predominantly negatively charged, with the exception of Gma_P21-like osmotin (Figure 4C).

### Gene Expression Data Mining

**FIGURE 5:**
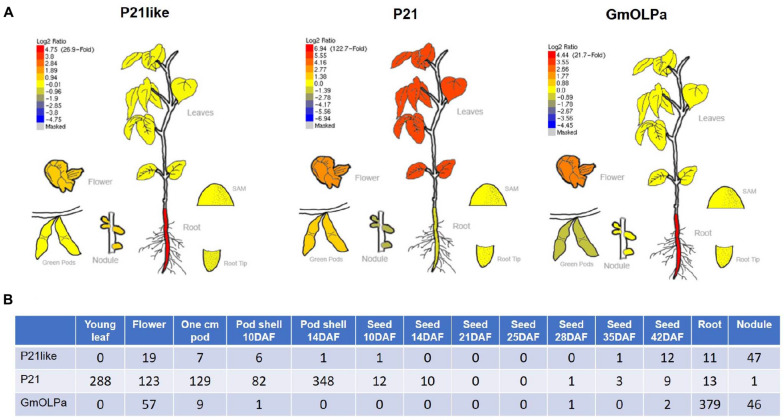
*In silico* gene expression analysis. **(A)** Relative expression profile of soybean osmotin genes from the RNA-seq BAR database (http://bar.utoronto.ca/efpsoybean/cgi-bin/efpWeb.cgi) (Libault et al., 2010). *Red* and *blue* colours indicate upregulated and downregulated genes, respectively (Log^2^ ratio) in different organs/tissues as plotted in the *bar* scale. **(B)** Digital gene expression counts of the uniquely mappable reads from RNA-Seq Atlas of *Glycine max* raw data (Severin et al., 2010). RNA-seq reads were only mapped to the initial genome assembly (i.e., Wm82.a1.v1).

In order to gain insights regarding soybean osmotins expression, RNA-seq data available from different sources were investigated. Among soybean osmotins, only the P21, P21-like, and GmOLPa relative expression was observed in the investigated sources (Figure 5 and Table 3). Using the Soybean eFP Browser, an up regulation of P21like and GmOLPa was detected in roots, while P21 up-regulation was detected in leaves. A slightly increased expression of the three genes was also observed in soybean flowers (Figure 5A). Using the RNA-Seq Atlas of *Glycine max*, a greater number of read counts in flowers was detected in the three genes, especially P21. P21 also exhibited a high number of read counts in young leaves and pods, while GmOLPa showed higher counts in roots. GmOLPa and P21-like were also expressed in nodules (Figure 5B).

**TABLE 3:** Microarray and RNA-seq data from soybean submitted to drought stress experiments.

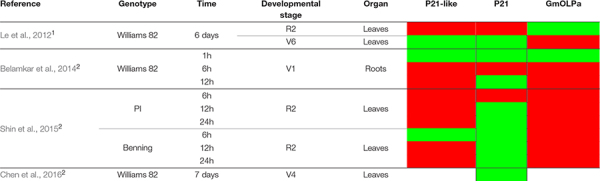

*^1^Microarray analysis.*

*^2^RNA-seq analysis.*

**Red* and *green* rectangles indicate *upregulation* and *downregulation*, respectively.*

Considering RNA-seq data, the differential expression of osmotin genes in response to dehydration and developmental stage has been verified (Table 3). P21 has shown to be upregulated in response to drought stress in leaves, during reproductive stage (R2), and 6 hours after treatment in the PI 416937 genotype (slightly sensitive to dehydration stress) (Shin et al., 2015) and 6 days after treatment in the Williams 82 cultivar (highly sensitive to drought) (Ha et al., 2015). Data from Belamkar et al. (2014) and Shin et al. (2015), showed that P21-like and GmOLPa osmotins were upregulated 6 and 12 hours after drought treatment in the roots of Williams 82 during the vegetative stage, and 6, 12, and 24 h after treatment in leaves of the PI 416937 and Benning cultivars (highly sensitive to dehydration stress) during the reproductive stage. According to Le et al. (2012) data, P21-like osmotin was upregulated and downregulated 6 days after drought treatment in the leaves of Williams 82 during the reproductive and vegetative stages, respectively. However, the response was the reverse for GmOLPa (Table 3).

### Physiological Analyses

Plants of the two soybean cultivars BR16 and EMB48 have been described as highly and slightly sensitive to dehydration stress (Oya et al., 2004), and were used in two different experiments to investigate soybean osmotin expression in response to drought stress.

**FIGURE 6:**
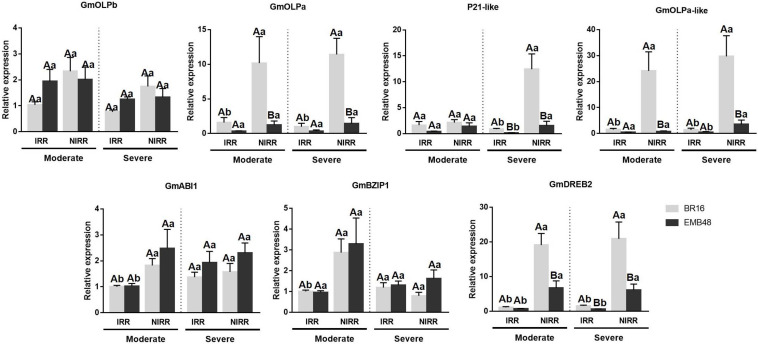
Gene expression analyses in a greenhouse experiment involving plants at the R1 development stage submitted to moderate and severe stress. The relative expression levels of soybean osmotin genes (GmOLPb, GmOLPa, P21-like and GmOLPa-like) and ABA response markers (GmABI1, GmBZIP1, and GmDREB2) in leaves were measured by RT-qPCR. NIRR, non-irrigated plants. Irrigated (IRR) plants of each cultivar were used as control. CYP2 and ELF1A reference genes were used as internal controls to normalise the amount of mRNA present in each sample. All transcript levels were calibrated in relation to the expression level of BR16 IRR plants under moderate water stress. Data represent the means of four biological replicates with three technical replicates each. Capital letters correspond to the comparison among means of cultivars within the same treatment. Small letters correspond to the comparison among means of treatments within the same cultivar. Means labelled with the same letter do not differ significantly (Duncan’s test, *p* < 0.05). Error bars represent the standard error of the mean.

In the first experiment physiological variables were verified to determine the plant stress status for posterior gene expression evaluation (Table 4 and Figure 6, respectively).

**TABLE 4:** Physiological variables of two soybean cultivars (BR16 and EMB48) at the R1 developmental stage under moderate and severe water stress.

Physiological variable	Stress	Treatment	BR16		EMB48	
RWC (%)	Moderate	IRR	86.3 (+ 4.5)	Ab	90.7 (+ 0.8)	Aa
		NIRR	71.7 (+ 5.0)	Ab	74.1 (+ 5.2)	Ab
	Severe	IRR	90.8 (+ 0.4)	Aa	91.1 (+ 0.9)	Aa
		NIRR	54.9 (+ 0.8)	Bb	70.6 (+ 3.6)	Ab
Ψ_*MIN*_ (MPa)	Moderate	IRR	−0.67 (+ 0.05)	Aa	−0.77 (+ 0.05)	Aa
		NIRR	−1.31 (+ 0.18)	Ab	−1.24 (+ 0.28)	Ab
	Severe	IRR	−0.73 (+ 0.03)	Aa	−0.70 (+ 0.05)	Aa
		NIRR	−1.95 (+ 0.27)	Ab	−1.59 (+ 0.23)	Ab
ΔT_*leaf* – air_ (°C)	Moderate	IRR	−2.57 (+ 0.79)	Ab	−1.83 (+ 0.97)	Ab
		NIRR	0.77 (+ 0.90)	Aa	0.67 (+ 1.07)	Aa
	Severe	IRR	−2.25 (+ 0.28)	Ab	−3.13 (+ 0.65)	Bb
		NIRR	0.74 (+ 0.64)	Aa	−0.66 (+ 0.35)	Ba
PSII quantum yield	Moderate	IRR	0.60 (+ 0.04)	Aa	0.53 (+ 0.05)	Aa
		NIRR	0.44 (+ 0.05)	Bb	0.64 (+ 0.03)	Aa
	Severe	IRR	0.66 (+ 0.01)	Aa	0.68 (+ 0.003)	Aa
		NIRR	0.50 (+ 0.06)	Bb	0.67 (+ 0.02)	Aa

*IRR, irrigated; NIRR, non-irrigated; RWC, relative water content; Ψ_*MIN*_, minimum leaf water potential; ΔT_*leaf* – air,_ leaf temperature in relation to air temperature; PSII, photosystem II. Capital letters correspond to the comparison among means of cultivars within the same treatment. Small letters correspond to the comparison among means of irrigation treatments within the same cultivar. Means followed by the same letter do not differ significantly (Duncan’s test, *p* < 0.05). Values between parentheses represent the standard error of the mean.*

Relative water content (RWC) and leaf water potential can be used to determine plant water status, and integrates the effects of several drought adaptive traits (Mir et al., 2012). In the present study, the greatest difference in RWC was observed in the severe water stress regime (Table 4). At this point, the irrigated (IRR) plants of both cultivars exhibited higher RWC compared to the non-irrigated (NIRR) plants. Moreover, EMB48 NIRR plants presented higher RWC than BR16 NIRR. Differences among treatments in the same cultivar under moderate and severe stress were detected for minimum leaf water potential (Ψ_*MIN*_). NIRR plants of both cultivars exhibited a decrease in leaf water potential, indicating their efforts to cope with water deficit.

Leaf temperature could also be indicative of plant stress (Martynenko et al., 2016). Plants of both cultivars exhibited increased leaf temperature in relation to air temperature (ΔT) under drought stress conditions. However, under severe stress, EMB48 NIRR plants exhibited a lower ΔT compared to BR16 NIRR plants (Table 4).

The quantum yield of photosystem II reinforces the differences between the two cultivars under drought stress. Under both stress conditions (moderate and severe), EMB48 plants responded better than BR16 plants, and did not present significant differences between IRR and NIRR plants.

### Gene Expression Analysis

The gene expression of soybean osmotins and ABA response markers genes were accessed in plants analysed in this first experiment. All transcript levels were calibrated in relation to the expression level of BR16 IRR plants under moderate stress. The expression profiles of osmotin genes were presented in Figure 6. No differences were observed for GmOLPb or P21-like osmotins under moderate stress. BR16 NIRR plants exhibited incremental expression of GmOLPa and GmOLPa-like under both stress conditions, while EMB48 NIRR exhibited an increment only in GmOLPa-like expression under severe stress. An increment of P21-like expression was observed in NIRR plants for both cultivars under severe stress. When differences were detected between cultivars, BR16 plants generally presented higher expression of osmotin encoding-genes than EMB48 plants. A very low relative expression of P21 was detected (data not showed).

The expression of three ABA response markers (GmABI1, GmBZIP1, and GmDREB2) increased in BR16 NIRR plants under moderate stress. Under this stress condition, no differences were observed between cultivars in the expression patterns of GmAB1 and GmBZIP1. However, in BR16 NIRR plants, the expression of GmDREB2 was greater than in EMB48 NIRR plants. An increment in the expression of GmAB1 and GmDREB2 was observed in EMB48 NIRR plants. Under severe stress, no differences were detected in GmAB1 and GmBZIP1 expression. Under this condition, the induction of GmDREB2 was observed for both cultivars in NIRR plants. BR16 NIRR plants presented higher expression of GmDREB2 than EMB48 NIRR plants under both moderate and severe stress levels.

**FIGURE 7:**
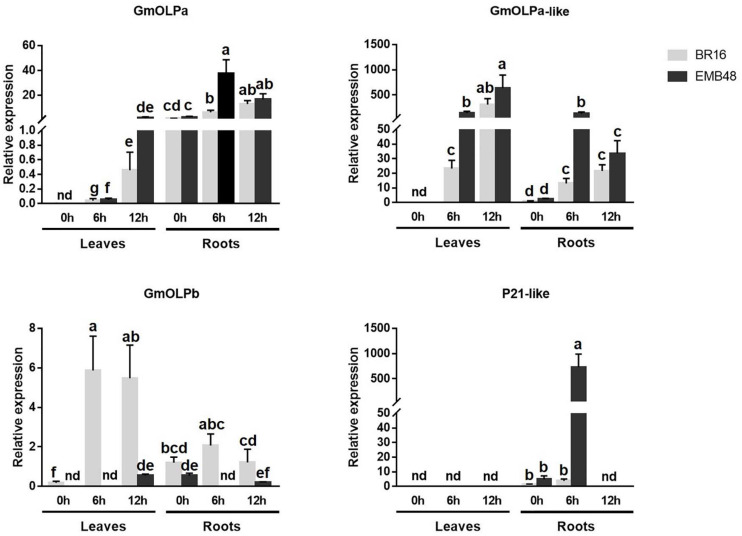
Gene expression analyses in a growth chamber experiment of plants at the V3 development stage submitted to drought stress. The relative expression levels of genes in leaves and roots were measured by RT-qPCR at 0 (control), 6, and 12 h after dehydration treatment. CYP2 and ELF1A reference genes were used as internal controls to normalise the amount of mRNA present in each sample. All transcript levels were calibrated in relation to the expression level of BR16 roots at 0h. Data represent the means of five biological replicates with three technical replicates each. Means labelled with the same letter do not differ significantly (Duncan’s test, *p* < 0.05). Error bars represent the standard error of the mean. Nd, not detected.

In the second experiment, BR16 and EMB48 plants were grown in a growth chamber until the V3 stage. At this stage, plants were removed from vermiculite and exposed to air at 0, 6, and 12 h. Leaves and roots were collected, and osmotin gene expression was evaluated (Figure 7). All transcript levels were calibrated in relation to the expression level of BR16 roots at 0h. At 0h, no expression was detected for osmotins in the leaves of both cultivars, except slight expression of GmOLPb in BR16. At the same time point, all genes presented expression in the roots of both cultivars. GmOLPa expression was regularly observed in roots of both cultivars. Its expression increased until 12h in leaves and until 6h in roots. The highest expression of GmOLPa was observed in EMB48 roots at 6h. The expression of GmOLPa-like also increased until 12h in leaves and until 6h in the roots of both cultivars. The highest level of GmOLPa-like expression was observed in EMB48 leaves at 12h, while EMB48 leaves and roots presented higher GmOLPa-like expression than BR16 at 6h. GmOLPb also presented higher expression in BR16 leaves at 6 and 12h. In EMB48, slight expression was detected for this gene in leaves at 12h and roots at 0 and 12h. P21-like osmotin expression was detected only in roots at 0 and 6h. The highest expression level for this gene was observed in the roots of EMB48 at 6h. No relative expression of P21 was detected (data not showed).

### *In silico* Promoter Analysis

Temporal and spatial gene expression is influenced by the presence of different *cis*-regulatory elements in the promoter region, where transcription factors can bind. The *cis*-elements analysis revealed a strong presence of binding motifs for AT-Hook and homeodomain transcriptional factors family (Table 5). Myb c*is*-elements were the third-most present among all soybean osmotin promoter regions. NAC and bZIP c*is*-elements were also well represented. Analysis of the available RNA-seq data (Le et al., 2012; Belamkar et al., 2014; Chen et al., 2016) from drought stressed soybean did not identify the upregulation of AT-Hook genes. However, homeodomain and Myb genes were among the most abundant upregulated transcription factors recorded under drought conditions.

**TABLE 5:** Transcription factor binding sites (TFBS) identified in the promoter sequences of soybean osmotin genes and the number of TFs upregulated in expression data.

	Number of *cis*-elements					Upregulated TFs in drought stressed soybean^1^		
		
TF family	GmOLPa	GmOLPa-like	GmOLPb	P21	P21-like	Le et al., 2012	Chen et al., 2016	Belamkar et al., 2014
**AP2**	8	3				17	19	35
**AP2; B3**	7	12	3	8	10			
**AT-Hook**	135	93	143	67	74	1		
**bHLH**	9	9	3	5	5	9	50	24
**bZIP**	13	27	23	33	21	9	32	9
**C2H2**	8	2	13	8	17	6	20	10
**bZIP; homeodomain; HD-ZIP**	2		4	2	2	3		
**EIN3**	6	5	3	8	11	2	2	
**GATA**	22	6	2	7	5		11	2
**Sox**	2		2	2	1			
**Homeodomain**	59	58	79	70	54	2	20	7
**MADF**	6	2	13	12	14		1	
**MADS box**	9	6	4	12	8	1	4	3
**Myb/SANT**	53	40	48	55	37	27	89	28
**NAC; NAM**	20	22	14	10	14	30	54	10
**SBP**	35	52	1	14	1	1		
**TBP**	25	47	83	40	25		4	
**TCP**	1	1	1	6		2	3	1
**TCR**	12	7	40	12	10			
**WRKY**	21	34	1	2	2	39	56	6
**CG-1**	2	6			2			
**GRAS**		2					13	5
**B3**			1		2		6	
**Motif sequence only**	82	96	75	99	68	-	-	-
**Others**	1	1		1	1	-	-	-

*^1^Available expression data with gene function annotation. Number of TFs upregulated genes according to each study.*

## Discussion

Various osmotin proteins have been identified from a variety of plants and characterised based on their potential subcellular location, p*I* value, and gene expression in response to biotic and abiotic stresses (Tachi et al., 2009; Chowdhury et al., 2015; Ullah et al., 2017). Furthermore, studies on transgenic plants overexpressing osmotins have demonstrated the potential of this overexpression to protect plants against different stresses (Weber et al., 2014; Kumar S. A. et al., 2016). Aiming to contribute to elucidate the roles of soybean osmotins in drought stress response the present study was carried out to reveal structural characteristics and expression pattern of four genes/proteins previously described and a novel putative soybean osmotin. *N. tabacum* and *S. nigrum* osmotins, previously characterised as providers of drought tolerance in plants, were used as references (Table 1). A phylogenetic tree was reconstructed and an osmotin clade was formed, thus allowing the identification of a novel soybean osmotin sequence (GmOLPa-like) (Figure 1 and Supplementary Figure 1). The GmOLPb soybean sequence was shown to be the most similar sequence to the Solanaceae osmotins, although it is a unique osmotin sequence that has two introns and no signal peptide (Figure 1A). In this context, Xu et al. (2012) highlighted that exon-intron structure alterations are prevalent in duplicated genes and, in many cases, have led to the generation of functionally distinct paralogs.

Duplication of genomic content can occur by many independent mechanisms, such as tandem duplication (consecutive duplications involving one or two genes), proximal duplications (duplications near one another but separated by a few genes), and whole-genome duplications (WGD; originate by polyploidy events) (Flagel and Wendel, 2009; Wang et al., 2012). GmOLPb previously characterised as a neutral osmotin (Tachi et al., 2009) was here classified as a tandem duplication found in chromosome 1 proximal to GmOLPa-like (Figure 1C), the novel putative osmotin sequence. GmOLPa-like is homologous to the acidic GmOLPa previously characterised by Onishi et al. (2006). The duplication pattern of GmOLPa-like, GmOLPa, and P21 was classified as WGD (Figure 1C). P21-like osmotin was located near P21 (its homologous sequence) on chromosome 5 and was classified as a proximal duplication. Duplicated genes can undergo neofunctionalisation (when one copy acquires a novel function) or subfunctionalisation (when both copies are mutated and adopt complementary functions) (Lynch and Conery, 2000; Lynch and Force, 2000).

Responses to stress generally involve integrated circuits involving multiple pathways and cellular compartments. The subcellular localisation of a protein can provide important information regarding its function within the cell (Abdin et al., 2011). The score location assignment program suggests that all osmotin sequences are secretory proteins (Figure 1A). Osmotins could also be synthesised as precursors presenting a C-terminal elongation that mediates their transport to the vacuole. This vacuole targeting was already identified for the *N. tabacum* and *S. nigrum* osmotins (Figure 1B; Campos et al., 2002). A C-terminal elongation was also identified in GmOLPb and proposed as vacuole targeting (Tachi et al., 2009). The acidic isoforms GmOLPa and P21 as well as P21-like and GmOLPa-like osmotins lack C-terminal elongation (Figure 1B). Osmotin proteins that lack C-terminal elongation have been predicted to be released into an extracellular space by the direct effects of the N-terminal signal peptide (Onishi et al., 2006). The secretory nature and multiple location targeting of osmotins are in agreement with their multifunctional role in plant responses to biotic and abiotic stresses (Abdin et al., 2011). Furthermore, it has been also demonstrated that both intracellular and extracellular osmotins may be involved in plant drought tolerance (Onishi et al., 2006; Parkhi et al., 2009). According to Chowdhury et al. (2017), higher concentrations of glycine, proline, and threonine in the interior of the cell might play an important role in the control of abiotic stress. However, in the present study, no significant differences in these amino acid contents among osmotin sequences were observed (Table 2).

The nine protein sequences here analysed (Nta_OSM, Sni_SnOLP, Sni_SindOLP, Sni_Jami, Gma_OLPa, Gma_GmOLPa-like, Gma_P21, Gma_P21-like, and Gma_OLPb) share the conserved amino acid signature and 3D structure of the thaumatin family, indicating their inclusion in this protein family (Figures 1B, 3). Despite their similarities, some differences were observed in the conserved regions of protein sequences and in electrostatic surface potential. The GmOLPa-like and GmOLPa sequences have two important substitutions involving amino acids with different properties in the acidic cleft region. These substitutions change a hydrophilic amino acid by a hydrophobic methionine^(210,216)^ in both sequences. GmOLPa-like also has an aspartate^109^ residue (D) instead of the glutamate^109^ residue (E) in the REDDD motif, which is an important sequence in the acidic cleft related to PR5 antifungal activity (Koiwa et al., 1999). According to the authors, in addition to its acidic nature, the cleft region is rich in hydrophilic residues, which is a characteristic fairly typical of carbohydrate-binding sites. Therefore, the acidic REDDD motif and the hydrophilic residues of the acidic cleft region are important in determining protein antifungal activity (Jami et al., 2007). In addition, a study conducted on the basic osmotin of tobacco suggests that the acidic cleft of this protein forms a hollow for Ca^+2^ electrostatic binding that facilitates the interaction to glycans on the surface of fungal cells, thereby leading to plasma membrane permeabilisation and damage (Salzman et al., 2004). Notably, as shown for some osmotins, Ca^+2^ is also related to enhanced plant drought tolerance by protecting the structure and stability of cellular plasma membranes against lipid peroxidation, elevating proline content, and maintaining normal photosynthesis (Song et al., 2008; Kumar et al., 2015). In addition, interactions between osmotins acid cleft charges and ion channels could improve the water influx through the plant cell membrane (Batalia et al., 1996). In spite of all nine analysed osmotins presenting a negative cavity, some differences in electrostatic potential were observed in the posterior region of the proteins and in their net charge. All soybean osmotins are negatively charged, except for P21-like, which has a positive net charge and a posterior region that is predominantly positively charged. The charge characteristics of P21-like are similar to those of *N. tabacum* and *S. nigrum* osmotins (Table 2 and Figure 4). Electrostatics potential plays an important role in molecular biology since it contributes to protein folding and stability, protein-protein interactions, ion binding, dimerization, protein-DNA/RNA interactions, and protein-microtubule binding (Shashikala et al., 2019). In particular, it is well-known that molecular electrostatics can be predictive of a molecule’s chemical reactivity and its ability to form certain types of interactions (Rathi et al., 2020). Differences in the topology and surface electrostatic potential surrounding the cleft are thought to determine the specificity of TLPs to their target proteins and ligands (Min et al., 2004).

The two cultivars BR16 and EMB48 - highly and slightly sensitive to dehydration, respectively - were evaluated for physiological variables to determine plant stress status. Differences between the two cultivars were observed for RWC, ΔT_*leaf* – air_, and quantum yield of photosystem II (Table 4). EMB48 retained more water in its leaves than BR16, maintaining a lower leaf temperature and photosystem II integrity, thereby reinforcing its characterisation as slightly sensitive to dehydration. A previous study showed that drought tolerant soybean genotypes were able to maintain RWC values and chlorophyll content at steady-state levels, even under stress conditions (Hossain et al., 2014). These physiological adaptive traits are frequently associated with abscisic acid (ABA) phytohormone signalling (Mir et al., 2012; Hossain et al., 2014; Martynenko et al., 2016). The results presented in the current study indicated that GmDREB2 ABA marker gene expression generally increased in both BR16 and EMB48 cultivars under moderate and severe water stress (Figure 6). GmDREB2 was previously described as being responsive to ABA signalling and being involved in ABA-dependent signal pathways in soybean (Chen et al., 2007). According to the authors, GmDREB2 acts as an important transcriptional activator and may be useful in improving plant tolerance to abiotic stresses. It has also been revealed that tobacco osmotin is induced in cultured cells and roots in response to ABA treatment and under polyethylene glycol (PEG)-mediated water or salt stresses (Ullah et al., 2017). According to Onishi et al. (2006), the expression of GmOLPa osmotin in response to ABA and dehydration may be primarily induced via an ABA-independent transcriptional pathway. The *in silico* promoter analysis of osmotins performed in the present study revealed a strong presence of AT-Hook, homeodomain, and Myb *cis*-elements (Table 5). The presence of Myb *cis*-elements (involved in dehydration and abscisic acid (ABA) response) upstream to the GmOLPa coding sequence was also reported by Onishi et al. (2006). Although NAC and bZIP *cis*-elements have not been as numerous in the promoter region of soybean osmotins, their encoding genes have been frequently identified as being upregulated under drought stress conditions. Notably, the GmOLPa-like and GmOLPa promoter region also have a small number of WRKY *cis*-elements. Moreover, the upregulation of WRKY-encoding genes has been related to drought stress response (Dias et al., 2016).

As previously mentioned, the overexpression of osmotins could also promote abiotic stress tolerance in transgenic plants (Ahmed et al., 2013). The present study demonstrated that soybean osmotins (GmOLPa, GmOLPb, P21-like, and GmOLPa-like) were differentially expressed in different organs (leaves and roots), developmental stages (V3 and R1), cultivars (BR16 and EMB48), and in response to dehydration (Figures 6, 7). In the first experiment, in which the leaves of soybean cultivars were collected at the R1 developmental stage, BR16 osmotins were more induced compared to EMB48 osmotins (Figure 6). However, in the second experiment, in which soybean plants were sampled at V3 stage, the GmOLPa, GmOLPa-like, and P21-like osmotins of EMB48 exhibited higher expression than those of BR16 at certain time points and organs (Figure 7). The expression of these three osmotins at 0h (control) was exclusive to roots. GmOLPa and GmOLPa-like exhibited expression in leaves following dehydration treatment, while P21-like osmotin showed expression only in roots (Figure 7). These results are congruent with *in silico* data that supports the expression of P21-like and GmOLPa osmotins being observed only in the roots of non-stressed soybean plants (Figure 5A). Onishi et al. (2006) also reported that dehydration for 24 h markedly increased expression of the GmOLPa gene in roots, and also induced low levels of expression in stems and leaves.

In conclusion, in the present work we characterized a new soybean osmotin-encoding gene (GmOLPa-like) and its expression pattern and putative product were compared to the already known osmotin isoforms (P21, GmOLPb, GmOLPa and P21-like). Our results show that the soybean osmotins expression pattern is organ and developmental stage dependent. The highest level of gene expression was detected for GmOLPa-like and P21-like osmotins in leaves and roots, respectively, of the less drought sensitive cultivar. We have also demonstrated that P21-like osmotin presents the most similar net charge to those osmotins previously characterised as promoters of drought tolerance in *N. tabacum* and *S. nigrum*. Overall, the results suggest the involvement of GmOLPa-like and P21-like osmotins in drought stress tolerance.

## Data Availability Statement

The original contributions presented in the study are included in the article/Supplementary Material, further inquiries can be directed to the corresponding author/s.

## Author Contributions

GF and MB-Z did the Study design. GF did the *in silico* analysis. FG did the duplication pattern analysis. PC, LT, and OS did the bioinformatic sequences analysis and comparative modelling. GF, DF, and CB did the soybean dehydration assays. DF and CB did the physiological analysis. GF and CR did the gene expression analysis. CB did the statistical analysis. GF, LT, and CB did the manuscript. LO-B did the manuscript revision. MB-Z did the study supervision and coordination. All authors read and approved the final manuscript.

## Conflict of Interest

The authors declare that the research was conducted in the absence of any commercial or financial relationships that could be construed as a potential conflict of interest.

## Footnotes

1 http://tree.bio.ed.ac.uk/software/figtree/

2 http://www.cbs.dtu.dk/services/SignalP/

3 http://www.cbs.dtu.dk/services/TargetP/

4 http://bar.utoronto.ca/efpsoybean/cgi-bin/efpWeb.cgi

5 http://frodo.wi.mit.edu/

6 http://plantpan2.itps.ncku.edu.tw/

## Supplementary Material

Supplementary Figure 1 Phylogenetic tree of 59 thaumatin domain sequences and gene structures from *G. max* and previously characterised *N. tabacum* and *S. nigrum* osmotins. Osmotin group is *pink* coloured according to posterior probability values.

Supplementary Table 1 Primer set designed for RT-qPCR.
