# Supplementary figures and images for: Molecular Characterisation of Soybean Osmotins and Their Involvement in Drought Stress Response
Source: Front Genet. 2021 Jun 25;12:632685. doi: 10.3389/fgene.2021.632685 (PMC8267864; doi:10.3389/fgene.2021.632685)

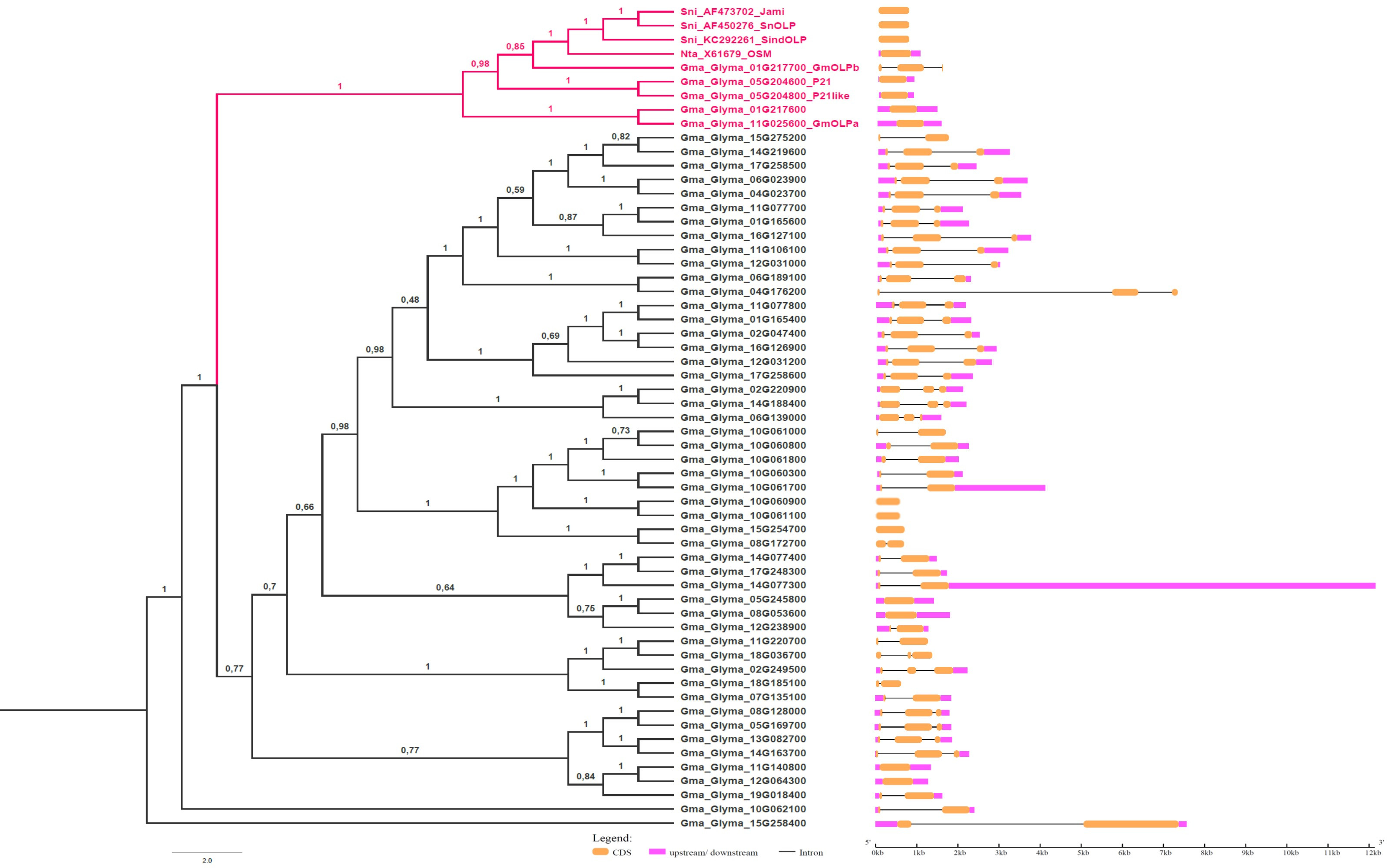

Supplement: Supplementary Figure 1 — Phylogenetic tree of 59 thaumatin domain sequences and gene structures from G. max and previously characterised N. tabacum and S. nigrum osmotins. Osmotin group is pink coloured according to posterior probability values. [file Image_1.JPEG]
